# Factors Contributing to Sharp Waste Disposal at Health Care Facility Among Diabetic Patients in North-East Peninsular Malaysia

**DOI:** 10.3390/ijerph16132251

**Published:** 2019-06-26

**Authors:** Ummu Atiyyah Hasan, Suhaily Mohd Hairon, Najib Majdi Yaacob, Aziah Daud, Anees Abdul Hamid, Norzaihan Hassan, Mohd Faiz Ariffin, Lau Yi Vun

**Affiliations:** 1 Department of Community Medicine, School of Medical Sciences, Universiti Sains Malaysia, Kubang Kerian, Kota Bharu, Kelantan 16150, Malaysia; ummumardiyyah@gmail.com (U.A.H.); aziahkb@usm.my (A.D.); 2 Unit of Biostatistics & Research Methodology, School of Medical Sciences, Universiti Sains Malaysia, Kubang Kerian, Kota Bharu, Kelantan 16150, Malaysia; najibmy@usm.my; 3 Primer Unit, Kelantan State Health Department, Kota Bharu, Kelantan 15200, Malaysia; dranees@moh.gov.my; 4 Kota Bharu District Health Office, Kelantan State Health Department, Kota Bharu, Kelantan 15200, Malaysia; norzaihanhassan@yahoo.com.my (N.H.); fransisca_007@hotmail.com (L.Y.V.); 5 Non-Communicable Disease Control Unit, Kelantan State Health Department, Kota Bharu, Kelantan 15200, Malaysia; drmohdfaiz@moh.gov.my

**Keywords:** diabetes, sharp waste disposal, health facility

## Abstract

Background: Type 2 diabetic patients are major users of medical sharps in the community. Proper sharp disposal practice among them, however, was reported to be low. The current study was aimed to determine the factors contributing to sharp waste disposal at a health care facility among Type 2 diabetic patients. Methods: In this cross-sectional study, Type 2 diabetic patients who were on insulin therapy attending health clinics were randomly selected and interviewed using a validated questionnaire. Binary logistic regression analysis was applied. Results: Out of 304 respondents, only 11.5% of them brought their used sharps to be disposed at health care facilities. Previous advice on sharp disposal from health care providers, knowledge score, and duration of diabetes were significant contributing factors for sharp waste disposal at health care facilities: (Adj. OR 6.31; 95% CI: 2.63, 15.12; *p* < 0.001), (Adj. OR 1.05; 95% CI: 1.03, 1.08; *p* < 0.001), and (Adj. OR 2.51; 95% CI: 1.06, 5.93; *p* = 0.036), respectively. Conclusion: Continuous education and a locally adapted safe sharp disposal option must be available to increase awareness and facilitate diabetic patients adopting proper sharp disposal behavior.

## 1. Introduction

Diabetes mellitus (DM) is now becoming an important public health concern globally, with the number of adults living with diabetes being reported to be on an escalating trend. Of these, Type 2 DM accounts for the vast majority of people with diabetes, causing a significant public health burden [1]. The majority of this increase occurs in developing countries, including Malaysia, due to population growth, ageing, unhealthy diet, obesity, and sedentary lifestyle [2,3]. As the disease progress, patients with Type 2 DM may have worsening glycaemia due to a progressive decline in beta cell function. This will eventually cause oral anti-diabetic agents to be ineffective, thus, most of diabetic patients may require long-term insulin therapy [4]. Worldwide, the number of insulin-requiring diabetic patients is increasing, likewise in Malaysia [5,6].

Diabetic patients who are prescribed with insulin are advised to perform self-monitoring of blood glucose (SMBG) using glucometer and lancets to regularly monitor their blood glucose level and self-adjust insulin doses accordingly [7]. This process usually requires shifting to a more intensive insulin regimen with an increased number of injections to achieve better glycaemic control [8]. All these procedures then generate a considerable amount of sharp waste within the household setting. Because of the increasing prevalence and increasing use of insulin therapy and SMBG, diabetic patients, primarily Type 2 DM who are insulin-dependent, are identified as major users of medical sharps in the community [9].

While medical sharp handling and disposal in health care setting is well regulated, sharps generated in household and community settings, however, are not yet given much attention. This is of particular concern especially in many developing countries, where resources are constrained and waste disposal systems are very limited [10,11,12,13]. Nonetheless, in several developed countries like the United States (U.S.) and Australia, specific ordinance to regulate sharp disposal in residential settings are in place to cater for sharp waste generated for home treatment by all patients with chronic diseases, including diabetes [14,15].

Similar to other developing countries, while sharps at health care setting in Malaysia are strictly regulated, sharps generated at community settings has not been treated similarly yet. The same regulation is still not being mandated in household settings. There is a local guideline pertaining to community sharp disposal, but it is specifically aimed towards injecting drug users, which was implemented in a specific needle syringe exchange program [16]. However, until now, no guideline or community sharp disposal program has been available to handle sharps used by other chronic disease patients who require self-injecting medication, especially those with diabetes.

As proposed by the two established agencies, U.S. Food and Drug Administration (FDA) and U.S. Environmental Protection Agency (EPA), sharps must be discarded into a safe sharp container and subsequently disposed at any designated central collection areas such as a health care facility, pharmacy, medical waste facility, or household hazardous waste collection site, depending on local policies and regulations [14,17,18]. Other options of disposal include residential special waste pick-up service, home needle destruction devices, and mail-back service [19]. However, it is reported that only a relatively small proportion of diabetic patients managed to return the sharp wastes in proper containers to the central collection area for final disposal [12,13,20].

While there is a large amount of local literature on sharp disposal at healthcare setting available, to the best of researchers’ knowledge, so far, no local study had been conducted to assess sharp disposal practice among the Malaysian diabetic population. Therefore, this current study aimed to determine factors contributing to sharp waste disposal at health care facilities among Type 2 DM patients.

## 2. Materials and Methods

### 2.1. Study Setting and Participants

A cross sectional study was conducted among Type 2 DM patients attending government primary health clinics in two selected districts in Kelantan state, which is located in the North-East region of peninsular Malaysia. The two districts, Pasir Mas and Pasir Puteh, were selected for this study because both were among the districts which recorded the highest number of Type 2 DM patients in Kelantan [21]. Only government primary health clinics which had designated diabetic teams that provided primary management for Type 2 DM patients were considered in this study. The study was conducted within a two months duration, from February 2018 until March 2018. The study involved Type 2 DM patients who were registered at these primary health clinics. The inclusion criteria were; 1) Type 2 DM patients aged 18 years old or older [8], 2) had been using insulin for at least one-month duration, and 3) self-injected their insulin and/or self-tested their blood glucose levels. Those patients with gestational diabetes mellitus or established Type 2 DM in pregnancy and patients with mental illness were excluded from the study.

### 2.2. Sample Size Determination and Sampling Method

The number of participants required was calculated using power and sample size (PS) software [22]. Calculation option “dichotomous” for comparison of two independent proportions in the software was selected. This option is indicated to be used for sample size determination in studies which involve hypothesis testing of odds ratio (OR) between two independent groups. The null hypothesis (H_0_) was the odds of exposure in the group without outcome is equal to odds of exposure in the group with outcome, resulting in OR between the two group to be 1 (OR = 1). The proportion of exposure in the first group was obtained from a previous study (P_0_ = 0.31) [23] and an OR of 2.0 was decided to be the smallest OR to be detected, resulting in a small Cohen’s *h* effect size of 0.34. With type I error probability of 5%, type II error probability of 20% (resulting in 80% power of study), and a ratio between the groups of 1, the required sample size for this study was 278. Anticipating 10% dropout due to non-response or missing data, the final required sample size was 309. A multistage sampling was applied. In each of the selected Pasir Mas and Pasir Puteh districts, simple random sampling using computer generated software was used to select four government primary health clinics which had a diabetic team from each district. To select patients from each clinic, stratified sampling using probability proportional to size was conducted. The total number of registered Type 2 DM patients on insulin therapy in each health clinic was obtained from the non-communicable disease unit, Kelantan state health department. Patients in the two districts were then stratified according to health clinic where they received diabetes treatment, as each clinic had a different number of registered patients. The number of samples recruited from each clinic was proportionate to the actual number of available Type 2 DM cases. Then, systematic random sampling was conducted at each clinic on every data collection day. The first subject who fulfilled the inclusion and exclusion criteria was randomly chosen from the diabetic outpatient registration list on that specific day of data collection. The subsequent subjects were selected at regular two-unit intervals (4th, 6th, 8th) until the required sample size was achieved.

### 2.3. Data Collection

Participants were interviewed using two sets of questionnaires, a proforma, and a validated Malay version of diabetes community sharp disposal (M-DCSD) questionnaire. The interview was primarily conducted by the main researcher and a trained research assistant. A standardized interview was conducted for each respondent, which took about 10 to 20 min to be completed for each one of them.

#### 2.3.1. Proforma

A proforma was used to record the information on sociodemographic characteristics, diabetes, and diabetes treatment characteristics, and previous advice on sharp disposal from health care providers (HCPs), which where self-reported by the participants. For a more objective measurement, after the interview session ended, the researcher double checked the information regarding diabetes and diabetes treatment in participants’ diabetes treatment card.

#### 2.3.2. Malay Version of Diabetes Community Sharp Disposal (M-DCSD) Questionnaire

A validated questionnaire on community sharp disposal was used to collect data on knowledge and sharp disposal practice from the participants. The original version was developed by Quiwa and Jimeno [13] based on a health belief model. The knowledge domain measured three subdomains; proper sharp use, hazards of improper sharp disposal, and proper sharp disposal method. The domain was assessed with 10-item with 4-multiple choice response and a single best answer. True answer was given a ‘1’ score and false answer was given a ‘0’ score. The total score was converted to a percentage and would range from 0 to 100%. This domain had good psychometric properties, with a mean item difficulty index of 0.623, corresponding to a test of moderate difficulty. All the items had acceptable discrimination index and point-biserial, with values ranging from 0.22 to 0.43 and 0.31 to 0.53, respectively. The practice domain measured two subdomains; use of sharp container and methods of sharp disposal. It was assessed with three items with multiple choice response and more than two correct answers. Prior to the current study, the questionnaire was translated and culturally adapted into a Malay version [24]. The translated version was validated and has a good content validation index as rated by a team of field experts, with 0.92 for knowledge domain, and 1.00 for practice domain, respectively. Face validation index was 0.95 for knowledge domain, and 1.00 for practice domain, indicating that the questionnaire was easily understood by the targeted respondents. For knowledge domain, the mean item difficulty index was 0.604. All of the items had an acceptable discrimination index with values ranging from 0.24 to 0.45. Internal consistency by marginal reliability was determined to be 0.64.

### 2.4. Statistical Analysis

Data were analyzed using statistical program for social sciences (SPSS) version 24.0 software (IBM, Armonk, NY, USA). Numerical variables were presented as means and standard deviations (SD) for normally distributed data, or median and interquartile range (IQR) for skewed data, whereas categorical variables were presented as frequencies and percentages. To determine the factors contributing to sharp waste disposal at health care facilities, logistic regression analyses were performed. Simple logistic regression was used to select the preliminary variables that have an association with sharp disposal at health care facilities. Multiple logistic regression was used to identify the independent contributing factors. All variables were included in model building to obtain a preliminary main effect model. The preliminary main effect model was produced after comparing models using backward likelihood ratio and forward likelihood ratio methods. All possible two-way interactions and multicollinearity between variables were checked, to obtain a preliminary final model. The final model was established after confirmation of model fitness by checking Hosmer and Lemershow goodness of fit test, classification table, and receiver operating characteristic (ROC) curve. The level of significance was set at *p*-value of less than 0.05.

### 2.5. Ethical Consideration

The study was ethically approved by the human research ethics committee of Universiti Sains Malaysia (Code: USM/JEPeM/17110663) and the national medical research register (NMMR) Malaysia (Code: NMRR-17-2633-38683 (IIR)). All participants gave written consent to participate. The confidentiality of the data was strictly protected. All reporting and publication were carried out in complete anonymity with no respondents named.

## 3. Results

In this cross-sectional study, a total of 304 eligible Type 2 DM patients were recruited. All of them completed the survey and were included in the final analysis.

### 3.1. Socio-Demographic Characteristics of Type 2 Diabetic Patients on Insulin

The range of age of patients was between 19 years and 83 years with a mean of 57.01 (10.59) years. The majority of them were female (71.1%), Malay race (99.0%), unemployed or retired (62.8%), and had a secondary education background (54.3%). About one third of them (36.8%) had children at their place of residence. Table 1 shows the socio-demographic characteristics of Type 2 diabetic patients on insulin.

### 3.2. Diabetes and Diabetes Treatment Characteristics of Type 2 Diabetic Patients on Insulin

Most of the respondents had been diagnosed with DM for five years or more (80.3%). However, only 22.7% of them had been prescribed with insulin therapy within a duration of 5 years or more. The majority of them were on insulin with oral anti diabetic agents (72.7%), took an insulin injection twice daily (50.3%), used a reusable insulin pen (94.1%), and did SMBG at home (51.6%). Concomitant infectious diseases were also found in a small proportion, 7.0% for either Hepatitis B or Hepatitis C. Table 2 shows the diabetes and diabetes treatment characteristics of Type 2 diabetic patients on insulin.

### 3.3. M-DCSD Total Knowledge Score and Previous Advice on Sharp Disposal

The mean (SD) of M-DCSD total knowledge score was found to be 59.34 (20.25). Only 35.9% of respondents recalled ever being advised by HCPs on sharp use and disposal since being prescribed with insulin therapy.

### 3.4. Sharp Waste Disposal at Health Care Facility

Out of 304 respondents, only 35 (11.5%) patients managed to dispose their used sharps at a health care facility, either at health clinics or hospitals. The majority of them used non-heavy-duty containers (88.6%), especially plastic bags, to hold the sharps, and 8.6% of them mixed both sharps and non-sharps together. At health care facilities, most of them disposed of sharps at pharmacy counters (40.0%), while 2.9% of them disposed of sharps at the registration counter. The summary of the findings is displayed in Table 3.

### 3.5. Factors Contributing to Sharp Waste Disposal at Health Care Facility

From multiple logistic regression analysis, the significant independent factors contributing to sharp waste disposal at health care facilities were previous advice on sharp disposal from HCPs (Adj. OR 6.31; 95% CI: 2.63, 15.12; *p* < 0.001), M-DCSD knowledge score (Adj. OR 1.05; 95% CI: 1.03, 1.08; *p* < 0.001) and duration of diabetes (Adj. OR 2.51; 95% CI: 1.06, 5.93; *p* = 0.036), when other variables were controlled. A summary of all the contributing factors is presented in Table 4 and Table 5.

## 4. Discussion

Irrespective of the setting where they are being produced, medical sharps are recognized as highly hazardous clinical waste which requires proper collection, disposal, and complete destruction to prevent potential risk of injury or infection to any person coming into contact with it [9]. As in Malaysian context, clinical sharp waste is classified as scheduled waste that is regulated under the Environmental Quality Act 1974, which states that the waste needs to be disposed at a central collection area and destructed via incineration process [25]. The regulation was effectively being applied at all health care settings throughout the country [26], but not yet adequately implemented for sharps generated in a household setting.

### 4.1. Sharp Waste Disposal at Health Care Facility

In this study, only one fifth of Type 2 diabetic patients managed to bring and properly dispose sharps at health care facilities. Most of them, however, did not use suitable containers to hold their sharps. The majority used plastic bags, most likely because these items were readily available and easy to find in their household. However, all these containers did not fulfil the basic features of a good sharp disposal container, as proposed by FDA and EPA [17,18]. This pattern was paralleled with previous studies in other developing countries. In The Philippines and Africa, for instance, only 2.0% of their diabetic patients disposed sharps at health care facilities [12,13]. However, in developed countries like Turkey and France, about 24.0% to 26.0% of their patients managed to collect sharps in either FDA-cleared or safe household containers, then disposed of the containers at health care facilities or other designated central collection areas. The availability of local guidelines, user-friendly community sharp disposal programs, and proper education from health care providers facilitated them to adopt safe sharp disposal behavior [27,28]. Some patients in the current study mixed both sharps and non-sharps, forcing health staffs to manually segregate the waste before discarding them into the sharp bin. Some of them also disposed their sharps at improper places like the registration counter. All these further actions unnecessarily expose health staff to the risk of accidental needle injury upon handling the sharps.

### 4.2. Factors Contributing to Sharp Waste Disposal at Health Care Facility

Studies have shown that proper education by HCPs have improved self-management behaviors among diabetic patients [29]. In this current study, it is observed that diabetic patients who ever received advice on sharp disposal were six times more likely to bring their used sharps at health care facilities to be properly disposed, as compared to those who never received any advice before. This finding was in line with previous studies that identified that diabetic patients who had been advised and instructed on how to properly use and dispose sharps were more likely to do the same, than those who had never been advised [20,23]. Another study in India found that diabetic patients who received the information on sharp disposal from their physicians were less likely to discard the sharps directly into domestic trash, as compared to those who were never informed [30].

According to the health belief model, cues are needed to promote engagement in health-promoting behaviors. External cues or triggers which might include event or information from close people, and information from media or HCPs, would activate the readiness and stimulate overt behaviors, especially if the perceived threats and perceived benefits versus perceived barriers are already high [31,32]. Thus, it is hypothesized that specific education or advice related to community sharp disposal would act as cues or triggers for diabetic patients to promptly engage with proper sharp disposal practice, especially among patients who already believed that improperly disposed sharps would cause environmental pollution and injury to self and others, and believed that the risk would be substantially decreased by adopting safe sharp disposal behavior.

The findings of this recent study was contrary to the study by Quiwa and Jimeno [13] which discovered prior advice or education regarding sharp disposal had no effect on sharp disposal practice. It was postulated that the advice given to diabetic patients in their study might not be correct in the first place, because even HCPs themselves were not aware of any safe sharp disposal option in their community. In addition to that, despite being informed on proper ways to discard the sharps, patients themselves might not follow the advice given to them, thus resulting in a very low proportion of patients who practiced safe sharps disposal manner.

Effective diabetes self-management requires acquisition of subject-specific knowledge and skills for patients to engage with better self-care activities [29]. Knowledge directly enables diabetic patients to self-manage their behavior and influence their compliance later [33,34]. Concerning sharp disposal, patients’ knowledge is extremely needed to ensure safe disposal [11,19]. In this study, a significant association between knowledge and sharp disposal practice was observed. The higher the knowledge score, the higher the likelihood for the patient to return their used sharps at health care facilities for final disposal. This finding was coherent with a study done in Pakistan that found knowledge on risk for blood-borne disease transmission following needle stick injuries was associated with safe sharp disposal practice among their diabetic population [30]. Kotchen [35] reported patients who were aware on possible hazards following improper disposal were three times more likely to bring the waste to the designated collection center for final disposal.

Knowledge regarding safe sharp disposal is one of the required components for safe sharp disposal behavior. Knowledge acts as a mediating variable that interacts with other factors to affect health-related behavior [33,34]. Thus, diabetic patients would not return their used sharps at health care facilities unless they had at least a minimal level of health motivation to do so, and knowledge on possible hazards, as well as knowledge regarding safe sharp disposal options in their local setting. They also might see themselves as vulnerable and the condition as threatening to them and others, convinced of the efficacy of safe disposal behavior to minimize the risk and able to acknowledge and overcome the barriers, which enables them to adopt safe sharp disposal practice.

The duration of diabetes is one of the known risk factors for diabetes self-care practices [34,36]. In this current study, a significant association between duration of diabetes and sharp return at health care facilities was observed. Patients who had diabetes for less than five years had almost three times the odds to return the sharps at health care facilities, as compared to those with diabetes for five years or more. This result was supported by earlier studies which reported patients with longer duration of diabetes were less likely to discard the sharps in an appropriate manner [13,23].

In order to explain why patients with a longer duration of diabetes were more likely to adopt improper sharp disposal behavior, we need to look into the pathophysiology of diabetes and its relation to general body function. Diabetes is a chronic disease that may require life-long medication, treatment, and self-care. Because of the chronicity of the disease, diabetic patients who already had diabetes for a longer duration could experience diabetes burnout [37]. They could become exhausted and frustrated from the long-term use of medications and continuous self-management, thus they might gradually neglect their diabetic self-care [36]. This might also include the way they use and discard of their sharps as well. On top of that, with time, patients, especially those with poorly controlled diabetes, might progress into a more severe disease with various comorbidities and complications. Therefore, this poorer physical well-being with longer duration of diabetes might impair their diabetic-related self-management and self-care activities [34,37], including their sharp disposal behavior.

### 4.3. Study Limitations

The assessment of sharp disposal practice and previous advice on sharp disposal mainly used a self-reporting method. As such, inaccurate reporting might potentially arise following respondent and recall bias which might affect the internal validity of the study. Another threat to internal validity of this study was the possibility of interviewer bias. To minimize the influence of interviewer bias in this study, the interviewer-guided questionnaire was primarily conducted by the main researcher and a research assistant. The research assistant was adequately trained by the researcher to conduct the interview session. A standardized interview was conducted for each respondent to ensure that the differences in the answers given were not contributed to by the conduct of the interview. Despite these limitations, this study provided a valuable local data on sharp handling and disposal in a community setting. Such data might provide evidence and guidance for authorities and policy makers to make an initiative improvement regarding this issue.

### 4.4. Future Research

Further research is required to identify the best options for community sharp disposal in Malaysian local setting. It would also be important to consider the roles of cost and convenience of each possible sharp disposal option, to ensure compliance and sustainability later. An intervention study to formally assess the effect of education on sharp disposal behavior might also benefit our diabetic population. It is also important for HCPs to be aware regarding appropriate sharp disposal techniques and the availability of local options, since their lack of understanding on these topics could contribute to improper sharp disposal practice among diabetic patients. Thus, further studies to assess provider knowledge would be useful.

## 5. Conclusions

Only a small proportion of diabetic patients managed to dispose their sharp waste at health care facilities. The significantly independent factors contributing to sharp waste disposal at health care facilities among diabetic patients were prior advice on sharp disposal, higher knowledge level, and longer duration of diabetes. By knowing these factors, it emphasizes the need for developing evidence-based guidelines and legislation, education, and a safe sharp disposal program in a Malaysian local setting to facilitate patients adopting proper sharp disposal behavior.

## Figures and Tables

**Table 1:** Socio-demographic characteristics of Type 2 diabetic patients on insulin (*n* = 304).

Variables	Frequency (%)	Mean (SD) (Range)
Age (years)		57.01 (10.59) (19–83)
Sex		
Male	88 (28.9)	
Female	216 (71.1)	
Race		
Non-Malay	3 (1.0)	
Malay	301 (99.0)	
Marital status		
Single/Divorced/Widowed	61 (20.1)	
Married	243 (79.9)	
Education		
None/Primary level	111 (36.5)	
Secondary level	165 (54.3)	
Tertiary level	28 (9.2)	
Occupation		
Unemployed/Retiree	191 (62.8)	
Employed	113 (37.2)	
Monthly household income		
More than RM3000	39 (12.8)	
RM1000-RM3000	136 (44.7)	
Less than R1000	129 (42.4)	
Presence of children at home		
No	192 (63.2)	
Yes	112 (36.8)	

**Table 2:** Diabetes and diabetic characteristics of Type 2 diabetic patients on insulin (*n* = 304).

Variables	Frequency (%)	Median (IQR)
Duration of diabetes (years)		
5 years or more	244 (80.3)	10.00 (9.0) *
Less than 5 years	60 (19.7)	
Treatment of diabetes		
Insulin with oral anti diabetic agents	221 (72.7)	
Insulin only	83 (27.3)	
Duration of insulin use (years)		
5 years or more	69 (22.7)	3.33 (3.0) *
Less than 5 years	235 (77.3)	
Frequency of insulin injection daily		
Once	75 (24.7)	
Twice	153 (50.3)	
Thrice	18 (5.9)	
Four times	58 (19.1)	
Insulin injection form		
Disposable insulin pen	14 (4.6)	
Reusable insulin pen	286 (94.1)	
Both disposable and reusable insulin pen	4 (1.3)	
SMBG		
No	147 (48.4)	
Yes	157 (51.6)	
Concomitant Hepatitis B or C		
No	297 (97.7)	
Yes	7 (2.3)	

**Table 3:** Description for sharp waste disposal at health care facility (*n* = 35).

Description for Sharp Waste Disposal at Health Care Facility	Frequency (%)
Use of sharp container	
Placed in heavy-duty plastic container	4 (11.4)
Placed in plastic bag	23 (65.7)
Placed in mineral water bottle	5 (14.3)
Placed in metal container	2 (5.7)
Placed in glass bottle	1 (2.9)
Place of disposal	
Pharmacy	14 (40.0)
Emergency room	6 (17.0)
Laboratory	5 (14.3)
Diabetic triage counter	5 (14.3)
Procedure room	3 (8.6)
Registration counter	1 (2.9)
No specific place for disposal	1 (2.9)
Segregate sharp and non-sharp waste	
No	3 (8.6)
Yes	32 (91.4)

**Table 4:** Factors contributing to sharp waste disposal at health care facility among Type 2 diabetic patients, using simple logistic regression (n = 304).

Variables	Frequency (%)		Crude OR (95% Cl)	Wald Statistic (df)	*p*-Value
	Disposed at Health Care Facilities (*n* = 35)	Not Disposed at Health Care Facilities (*n* = 269)			
Age (years)	52.97 (12.49) *	57.54 (10.22) *	0.96 (0.93, 0.99)	5.63 (1)	0.018
Sex					
Male	9 (25.7)	79 (29.4)	1		
Female	26 (74.3)	190 (70.6)	1.20 (0.54, 2.68)	0.20 (1)	0.654
Education					
None/Primary level	6 (17.1)	105 (39.0)	1		
Secondary level	24 (68.6)	141 (52.4)	2.98 (1.18, 7.55)	5.30 (1)	0.021
Tertiary level	5 (14.3)	23 (8.6)	3.80 (1.07, 13.54)	4.25 (1)	0.039
Occupation					
Unemployed/Retiree	16 (45.7)	97 (36.1)	1		
Employed	19 (54.3)	172 (63.9)	0.67 (0.33, 1.36)	1.23 (1)	0.268
Monthly household income					
More than RM3000	10 (28.6)	29 (10.8)	1		
RM1000-RM3000	13 (37.1)	123 (45.7)	0.31 (0.12, 0.77)	6.37 (1)	0.012
Less than R1000	12 (34.3)	117 (43.5)	0.30 (0.12, 0.76)	6.50 (1)	0.011
Presence of children at home					
No	24 (68.6)	168 (62.5)	1		
Yes	11 (31.4)	101 (37.5)	0.76 (0.36, 1.62)	0.50 (1)	0.481
Duration of diabetes					
5 years or more	21 (60.0)	223 (82.9)	1		
Less than 5 years	14 (40.0)	46 (17.1)	3.23 (1.53, 6.82)	9.47 (1)	0.002
Duration of insulin use					
5 years or more	6 (17.1)	63 (23.4)	1		
Less than 5 years	29 (82.9)	206 (76.6)	1.48 (0.59, 3.72)	0.69 (1)	0.407
Concomitant Hepatitis B or C					
No	33 (94.3)	264 (98.1)	1		
Yes	2 (5.7)	5 (1.9)	3.20 (0.60, 17.16)	1.84 (1)	0.175
SMBG					
No	14 (40.0)	133 (49.4)	1		
Yes	21 (60.0)	136 (50.6)	1.47 (0.72, 3.01)	1.10 (1)	0.295
Previous advice on sharp disposal from HCPs					
No	8 (22.9)	187 (69.5)	1		
Yes	27 (77.1)	82 (30.5)	7.70 (3.35, 17.66)	23.19 (1)	<0.001
M-DCSD knowledge score	76.29 (17.34) *	57.14 (19.58) *	1.06 (1.04, 1.09)	23.79 (1)	<0.001

*Mean (SD).

**Table 5:** Factors contributing to sharp waste disposal at health care facility among Type 2 diabetic patients, using multiple logistic regression (*n* = 304).

Factors	B	Adjusted OR (95% Cl)	Wald Statistic (df)	*p*-Value
Previous advice on sharp disposal from HCPs				
No		1		
Yes	1.841	6.31 (2.63, 15.12)	17.01 (1)	<0.001
M-DCSD knowledge score	0.052	1.05 (1.03, 1.08)	16.23 (1)	<0.001
Duration of diabetes				
5 years or more		1		
Less than 5 years	0.92	2.51 (1.06, 5.93)	4.41 (1)	0.036

Backward LR method was applied; No multicollinearity and no interaction were found; Hosmer Lemeshow test, *p*-value = 0.124; Classification table 89.8% correctly classified; Area under Receiver Operating Characteristics (ROC) curve was 84.6%.
